# Sociability deficits after prenatal exposure to valproic acid are rescued by early social enrichment

**DOI:** 10.1186/s13229-018-0221-9

**Published:** 2018-06-14

**Authors:** Marcos Campolongo, Nadia Kazlauskas, German Falasco, Leandro Urrutia, Natalí Salgueiro, Christian Höcht, Amaicha Mara Depino

**Affiliations:** 1 Universidad de Buenos Aires, Facultad de Ciencias Exactas y Naturales, Departamento de Fisiología, Biología Molecular y Celular, Buenos Aires, Argentina; 2 CONICET-Universidad de Buenos Aires, Instituto de Fisiología, Biología Molecular y Neurociencias (IFIBYNE), Buenos Aires, Argentina; 3 FLENI, Centro de Imágenes Moleculares, Laboratorio de Imágenes Preclínicas, Buenos Aires, Argentina; 4 Facultad de Farmacia y Bioquímica, Cátedra de Farmacología, Universidad de Buenos Aires, Buenos Aires, Argentina; 5 UBA-CONICET, Ciudad Universitaria, Int. Guiraldes 2160, Pabellon 2, Ciudad de Buenos Aires, Argentina

**Keywords:** Autism spectrum disorder, Sociability, Piriform cortex, Dopamine

## Abstract

**Background:**

Autism spectrum disorder (ASD) is characterized by impaired social interactions and repetitive patterns of behavior. Symptoms appear in early life and persist throughout adulthood. Early social stimulation can help reverse some of the symptoms, but the biological mechanisms of these therapies are unknown. By analyzing the effects of early social stimulation on ASD-related behavior in the mouse, we aimed to identify brain structures that contribute to these behaviors.

**Methods:**

We injected pregnant mice with 600-mg/kg valproic acid (VPA) or saline (SAL) at gestational day 12.5 and evaluated the effect of weaning their offspring in cages containing only VPA animals, only SAL animals, or mixed. We analyzed juvenile play at PD21 and performed a battery of behavioral tests in adulthood. We then used preclinical PET imaging for an unbiased analysis of the whole brain of these mice and studied the function of the piriform cortex by c-Fos immunoreactivity and HPLC.

**Results:**

Compared to control animals, VPA-exposed animals play less as juveniles and exhibit a lower frequency of social interaction in adulthood when reared with other VPA mice. In addition, these animals were less likely to investigate social odors in the habituation/dishabituation olfactory test. However, when VPA animals were weaned with control animals, these behavioral alterations were not observed. Interestingly, repetitive behaviors and depression-related behaviors were not affected by social enrichment. We also found that VPA animals present high levels of glucose metabolism bilaterally in the piriform cortex (Pir), a region known to be involved in social behaviors. Moreover, we found alterations in the somatosensory, motor, and insular cortices. Remarkably, these effects were mostly reversed after social stimulation. To evaluate if changes in glucose metabolism in the Pir correlated with changes in neuronal activity, we measured c-Fos immunoreactivity in the Pir and found it increased in animals prenatally exposed to VPA. We further found increased dopamine turnover in the Pir. Both alterations were largely reversed by social enrichment.

**Conclusions:**

We show that early social enrichment can specifically rescue social deficits in a mouse model of ASD. Our results identified the Pir as a structure affected by VPA-exposure and social enrichment, suggesting that it could be a key component of the social brain circuitry.

**Electronic supplementary material:**

The online version of this article (10.1186/s13229-018-0221-9) contains supplementary material, which is available to authorized users.

## Background

Autism spectrum disorder (ASD) is a group of neurodevelopmental pathologies characterized by persistent deficits in social communication and restricted, repetitive behaviors and interests [1]. Symptoms appear in early childhood and persist throughout adulthood. Although both genetic and environmental causes have been proposed and tested [2], their etiology remains unknown. There is currently no consensus on the underlying neuropathology, and many brain structures have been claimed to play a relevant role in ASD symptoms, e.g., hippocampus [3, 4], prefrontal cortex [5], and cerebellum [3, 6].

While great strides have been made in the treatment of ASD, its effectiveness varies greatly depending on the case, and the underlying mechanisms of partially successful therapies are unknown [7]. In particular, clinical studies suggest that early social stimulation is the most effective treatment for autistic children, who show significant improvements in social behavior (e.g. [8–10]). Early social stimulation could counteract genetic and/or environmental risk factors, helping children to interact better with others [11].

There is extensive evidence on the effects of early environmental enrichment on brain development in animal models. Enrichment has been demonstrated to diminish the effects of genetic risk and injury on brain malfunction [12, 13], and environmental enrichment can revert many of the ASD-related behaviors in rodent models of autism [14, 15]. Our aim is to analyze the effects of early social stimulation (a specific component of environmental enrichment) on ASD-related behavior in a mouse model of ASD and to assess its consequences on brain function.

It has been extensively shown that prenatal exposure of mice to valproic acid (VPA) at gestational day 12.5 results in reduced social interaction in the adult male offspring [16] and increased stereotyped behaviors [17], and that these animals present several cellular and molecular alterations also observed in autistic individuals [16, 18]. In these studies, experimental mice exposed to VPA were weaned with other VPA-exposed mice.

To evaluate the contribution of the early social environment to the levels of sociability in adulthood, we weaned VPA mice in VPA only cages (VPA-VPA mice), or mixed them in cages containing animals prenatally exposed to VPA (VPA-SAL mice) and saline-treated animals (SAL-VPA mice) in 2:3 or 3:2 ratio. Mice interacted from postnatal day (PD) 21 to PD60 in their homecage. Given that we assume that social interactions are reciprocal after weaning, VPA-SAL animals, i.e., mice prenatally exposed to VPA who lived with control animals since PD21, received social stimulation from control animals, while VPA-VPA mice did not. These differences in social stimulation in the home cage could affect sociability in adulthood.

We next performed a battery of adult behavioral tests in order to compare VPA-VPA mice with VPA-SAL animals, to reveal if peers could rescue the behavioral phenotype caused by prenatal VPA exposure. In particular, we evaluated ASD-related behaviors and found that early social stimulation can revert the reduction in sociability observed in VPA-VPA mice in the social interaction test, but repetitive behaviors are increased in all VPA-exposed mice (evaluated in self-grooming and spontaneous alternation in the Y maze). The olfactory habituation/dishabituation test was performed to evaluate olfactory function and response to social odors, and VPA-VPA mice showed a specific deficit in social odors investigation. The novel object recognition test was performed to evaluate short-term memory and neophobia, two confounders in the social interaction test, and showed no differences between groups. We then analyzed anxiety- and depression-related behaviors since mood disorders have a high comorbidity with ASD [19] and can be affected by changes in social stimuli early in life, and found that VPA-exposed animals show less exploratory behaviors and increased depression-related behaviors.

After characterizing the behavioral alterations in our experimental groups, we analyzed brain glucose metabolism in an attempt to identify brain structures involved in these behavioral phenotypes. As our unbiased preclinical PET study pointed to altered function of the piriform cortex, we further analyzed this area and found changes in neuronal activity and dopaminergic function that are comparable to the alterations in sociability observed in VPA-VPA mice.

## Methods

### Animals and treatments

Outbred CrlFcen:CF1 female and male adult mice were obtained from the animal house at the Facultad de Ciencias Exactas y Naturales, University of Buenos Aires (Buenos Aires, Argentina). We chose this outbred strain because (1) it shows reliable intermediate levels of the behaviors analyzed, allowing the detection of increases and decreases in the behavioral parameters evaluated; (2) it has better breeding performance than inbred strains; and (3) it shows no VPA toxicity during pregnancy (neither litter size nor gestation time are affected). All animals were housed in the animal house on a 12:12 light to dark cycle and 18–22 °C temperature, with food and water ad libitum. All animal procedures were performed according to the regulations for the use of laboratory animals of the National Institute of Health (Washington, DC, USA) and approved by the institutional animal care and use committee of the Facultad de Ciencias Exactas y Naturales, University of Buenos Aires (CICUAL Protocol Nr. 6/2). Eight- to 10-week-old male mice were mated with nulliparous 8–10-week-old female mice. Females were controlled every morning, and the day when a vaginal plug was detected was considered the gestational day (GD) 0.5.

#### Prenatal treatment

On GD12.5, pregnant female mice were injected subcutaneously with either 600 mg/kg of valproic acid sodium salt (VPA; Sigma, St. Louis, MO, USA) in saline solution or with saline solution (SAL), and housed individually. The parturition day was registered as postnatal day 0 (PD0), and the cage bedding was not changed during the first postnatal week to avoid nest and maternal care alterations.

#### Postnatal treatment

On PD21, male pups prenatally exposed to VPA were weaned in cages containing four-five animals of the same treatment (VPA-VPA animals), or with control male pups (VPA-SAL animals) in cages containing two-three VPA-treated mice with three-two saline-treated mice (SAL-VPA animals). Control animals weaned with other control animals were denominated SAL-SAL animals (Fig. 2a). Mice interacted from PD21 to PD60 in their homecage. Littermates were assigned to different postnatal treatments (X-SAL and X-VPA), and offspring belonging to the same prenatal treatment group from different mothers were mixed at weaning. We performed all the studies on males because female offspring of VPA-injected dams does not show ASD-related behaviors (unpublished data; [17, 20]).

### Juvenile play

To evaluate sociability at weaning, an independent cohort of 16 VPA and 16 SAL animals was tested for juvenile play at PD21. We used an independent group to avoid interfering with the social enrichment process (postnatal treatment). Non-sibling mice from the same prenatal treatment were matched for similar body weights within 1-g difference. On PD20, one mouse in each pair was marked on its back with black marker to distinguish between animals during testing. On PD21, mice were isolated for 30 min and allowed to habituate to the testing room (10 lx). Each animal was then placed for a 10-min habituation period in the testing arena (floor: 30 cm × 30 cm of black PVC; walls: 30 cm high of black formic). Afterward, the pair of animals was placed again in the testing arena for 30 min, allowing them to interact freely while they were filmed. Testing was performed during the 2 h prior to the start of the dark phase (18:00 to 20:00 h) to maximize activity.

Behaviors were scored separately for each mouse in the pair. All behaviors were scored manually using keys and the video-tracking system ANY-maze (Stoelting, IL, USA) by an experimenter (M.C.) blind to treatment. We evaluated play solicitation (crawling and approaching events), investigative behaviors (events of anogenital sniffing, nose to nose sniffing or following), affiliative behaviors (time spent sitting side by side or in social grooming), and non-social behaviors (time spent exploring, self-grooming or sitting alone) as previously reported [21, 22].

### Adult behavioral testing

All adult behavioral tests were performed during the light period (between 10:00 and 17:00 h), with the exception of the Y-maze test that was performed during the 2 h before the start of the dark phase (18:00 to 20:00 h) to maximize exploration. For adult behavior, data from three independent cohorts are presented. Each cohort consisted of five or six litters per prenatal treatment. Mice were 8 weeks old at the beginning of testing, and all tests were separated by 1-week intervals to reduce any inter-test effect. Tests were performed in the order listed below (Fig. 2b). Mice were tested in a room next to the holding room. Previous to the beginning of each test, all animals were habituated to the illumination in the testing room for 30 min. After testing, each mouse was identified and placed in a holding cage until all animals from a cage had been tested. All the behavioral testing and manual scoring were performed by two experimenters (M.C. and N.K.) blind to treatment groups.

#### Social interaction test

The social interaction test was performed as previously described [4]. Briefly, animals were exposed to a 40 cm × 15 cm black rectangular arena divided in three interconnected chambers and placed under dim light (10 lx). A clear Plexiglass cylinder (7.5 cm of diameter, with several holes to allow for auditory, visual, and olfactory investigation between a test and stimulus mouse) was placed in each side of the compartment at the beginning of the test. Prior to the start of each test, one of the end chambers was randomly designated as the “non-social side” and the other as the “social side”. Animals were placed in the central compartment and allowed to explore for 5 min (habituation). Then, an unfamiliar, young (3 weeks of age) CF1 male mouse (social stimulus) was placed in one of the cylinders (social side), and an object (white, plastic 3-cm-tall cylinder) was placed in the other cylinder (non-social side). Social interaction was evaluated during a 10-min period. The time the subject spent sniffing the social stimulus or the non-social stimulus (nose inside a hole of the cylinder) was recorded manually using a key in the video-tracking system ANY-maze (Stoelting, IL, USA). The entire apparatus, including the cylinders, was cleaned with a 20% ethanol solution between tests to eliminate odors. The apparatus floor was covered with bedding to reduce the stress and was replaced after each test.

#### Elevated plus maze test

The elevated plus maze test (EPM) was performed as previously described [23]. The apparatus consisted of two open and two closed arms (open arms: 30 cm × 5 cm, 100 lx, surrounded by a 0.5-cm high border; closed arms: 30 cm × 5 cm, 43 lx, surrounded by 19 cm high walls), both elevated 50 cm above the floor. The walls and the floor were made of black PVC. Mice were placed into the central platform of the maze (5 cm × 5 cm, 100 lx) facing towards an open arm and allowed to explore the maze for 5 min. Locomotion data were collected by a video-tracking system (ANY-maze, Stoelting). Measured locomotor parameters were time spent in open arms, time in closed arms, time in the central platform, traveled distance in both open and closed arms, % distance in the open arms, and total traveled distance. Ethological parameters were scored manually during each session: number of rearings, grooming time, number of head dippings, and number of protected head dippings—which consisted in head dippings specifically performed in the center of the maze. The entire apparatus was cleaned with a 20% ethanol solution between tests to eliminate odors. For statistical analysis of this test, we selected uncorrelated variables (|*r*| < 0.7): the time spent in the open arms, the time spent in the closed arms, the time spent in the central platform, the distance traveled in closed arms, number of rearings, grooming time and number of both head dippings and protected head dippings.

#### Open field test

The open field test (OF) was performed as previously described [23]. Mice were placed in the arena (floor: 45 cm × 45 cm of black PVC; walls: 30 cm high of black formic; 100 lx) for 30 min. Animals were initially placed along one side of the arena, and the center region was defined as the central 23 cm × 23 cm area. Locomotion data were collected by a video-tracking system (ANY-maze, Stoelting). Measured behavioral parameters were time spent in the center, time spent in the border, distance traveled in the center, distance traveled in the border, % distance in the center, and total distance traveled. Grooming time and the number of rearings were scored manually during each session. For statistical analysis of this test, we selected uncorrelated variables (|*r*| < 0.7): total distance traveled, time spent in the center, time grooming, and number of rearings.

#### The olfactory habituation/dishabituation test

Olfactory discrimination was investigated using a slightly modified habituation/dishabituation protocol [24]. Each mouse was isolated in the testing cage (floor: 27 cm × 16 cm, height 12 cm) and habituated to a non-odorant cotton tip for 30 min. Then, animals were given three 2-min presentations of each odor: water, two non-social odors, and two social odors. Non-social odors were imitation vanilla and banana extracts, and social odors were male and female cage swipes. Odors were presented using a cotton tip with a 1-min inter-trial interval, which is the amount of time needed to change the cotton tip. The testing room was illuminated with 50 lx. The time spent sniffing the cotton tip was recorded manually using a key of the ANY-maze software (Stoelting). Animals that did not explore any of the presentations of an odor were excluded from the analysis (3 SAL-VPA, 2 VPA-SAL, 3 VPA-VPA).

#### Novel object recognition test

The novel object recognition test (NOR) was modified from a previous study [25]. Animals were first habituated to the experimental box (floor: 30 cm × 30 cm of black PVC; walls: 30-cm high of black formic; 10 lx) during 5 min and then placed individually in a holding cage during 5 min. After that, animals were placed again in the experimental box containing two identical objects, allowing them to explore the objects during a 5-min trial (training session). Animals were placed again in their cage for a 5-min period, to generate new short-term memories. Finally, animals were placed again in the experimental box, allowing them to explore two different objects (testing session) for 5 min. One of the objects was identical to those explored during the training session (familiar object), and the other was a different object (novel object). The objects were presented in the same locations as in the training session. The location of the novel object was randomly assigned (left or right) for each animal to avoid place preference during the testing session. The objects used were a small transparent glass Erlenmeyer or a 1.5-ml Eppendorf tube of similar size. The time spent sniffing each object was scored manually using keys in the ANY-maze software (Stoelting). The objects and the experimental box were cleaned with 20% ethanol solution between animals to eliminate olfactory cues. One VPA-SAL animal was removed from the analysis because it did not explore any of the objects during the test.

#### Self-grooming test

Mice were scored for spontaneous grooming behaviors as described earlier [4, 21]. Each mouse was placed individually into a clear Plexiglass cylinder (20 cm high, 5.5 cm wide), illuminated at 10 lx. Before testing, animals were habituated to the test cylinder in the testing room, for 1 h in two consecutive days. On the testing day, each mouse was scored during 10 min for cumulative time spent grooming all body regions, using a key of the ANY-maze software (Stoelting).

#### Light-dark box test

The light-dark (LD) test was performed as previously described [26]. A 45 cm × 45 cm arena was divided in half with an inverted black box (lit side: 100 lx; dark side: 1 lx). Animals were able to cross from one compartment to the other through a small hole in the wall (12-cm height, 8-cm width). Each mouse was placed under the hole facing the illuminated side of the box and tracked for 5 min using the ANY-maze software (Stoelting). Behavioral parameters analyzed were time spent in the lit compartment and distance traveled in the lit compartment.

#### Tail suspension test

The tail suspension test (TST) was performed as previously described [26]. Animals were suspended in the air using adhesive tape wrapped around the subject’s tail (about 4/5 from the base) and fixed to a wire at 25 cm of height from a wooden surface. This test was performed under 50 lx. The immobility time was scored during 5 min, using a key of the ANY-maze software (Stoelting). One animal was removed from this test because it learned to climb its tail.

#### Forced swimming test

The forced swimming test (FST) was performed as previously described [26]. Animals were gently placed in a beaker glass (15 cm in diameter and 25 cm in height), filled with 14 cm of water at 25 °C and illuminated with 50 lx. Immobility time was scored during 6 min using a key in the ANY-maze software (Stoelting). At the end of the test, animals were dried with a paper towel and placed into a holding cage.

#### Y maze test

The Y-maze was constructed of Plexiglas with three identical arms (42-cm long, 12-cm tall walls, 30 lx), and visual cues were located on the walls outside the maze. The maze floor was made of black PVC. One of the three arms was arbitrarily designated as the start and exploration was recorded using the ANY-maze software (Stoelting). Total locomotion and percentage of alternation are reported. Percentage of alternation was calculated as (alternations × 100)/(Total arm visits− 2), where an alternation was considered as consecutively visiting the three arms. One VPA-SAL animal was excluded because it performed less than fifteen alternations.

### Pre-clinical PET imaging

#### Imaging system

Images were acquired using a preclinical PET TriFoil LabPET 4 with LYSO and GYSO crystals and 1536 APD detectors groups. Approximated spatial resolution FWHM = 1.2 mm (*full width at half maximum*), 3.7 cm axial and 11 cm trans-axial FOV (*field of view*).

#### Animal handling

Pre-clinical PET imaging was performed on 15–17 animals for each experimental group, from two independent cohorts. Animals were starved during 18 h and then injected with 25 μCi/gr of [18F]-FDG i.p. and left undisturbed in an individual temperature-controlled (29 °C) cage for 30 min during radiopharmaceutical incorporation. Mice were then anesthetized using a mixture of isoflurane and O_2_ (inhalation, 4.5% induction and 1.5% maintenance dose) and maintained in a warm table (35 °C) during the acquisition.

#### Acquisition and reconstruction setup

Each subject was acquired for 12 min using list-mode acquisition. Images were reconstructed using an OSEM 3D algorithm with 30 iterations, to maximize SNR (*signal-to-noise ratio*). If motion was detected during acquisition, a dynamic reconstruction was performed in order to correct it using SPM5 on MATLAB® realign algorithm.

#### Image spatial processing

The images were confined to a bounding-box that only includes the brain. A normal subject-based template was created in order to have an anatomic reference for realignment and normalization. All subjects were smoothed using an isotropic Gaussian kernel with 1 mm FWHM. All images were co-registered and normalized to this template using SPM5 on MATLAB®, according to these parameters: Normalized mutual information as objective function and 7-mm smoothing histogram for rigid co-registration; and affine regularization to the averaged template size, no-smooth and 2–0.1 mm of separation for the non-rigid normalization. Previous to intensity normalization and statistical analysis, a brain masking avoiding Harderiand glands was applied to all subjects since the uptake in these glands can significantly modify the intensity normalization values.

#### Image statistical analysis

All subject groups were analyzed as a full-factorial ANOVA test using SPM5 on MATLAB®. Intensity normalization was considered as a regressor variable for each factor using grand mean scaling (ANCOVA). Global calculation of individual means was calculated over each masked brain. Parametric statistical images were calculated for all possible experimental group contrasts. In order to correct for multiple comparisons, false discovery rate (FDR) approach was applied using SPM5 (*p* value FDR: 0.05). In order to have an accurate anatomical reference, all results of statistical differences where co-registered with an MRI atlas [27]. Spatial transformation was applied to the MRI atlas to correct for the differences between mice strains and methodological animal handling.

### Catecholamine determination

An independent group of animals (*N* = 4–5 animals per group) was used for catecholamine determination as previously described [28]. Mice were sacrificed via cervical dislocation and punches of piriform cortex were taken and quickly frozen and kept at − 80 °C. Tissue was homogenized in 1 ml of 0.3 M perchloric acid, centrifuged for 15 min at 3000 g at 4 °C and then frozen at 80 °C. Levels of DA, DOPAC, 5-HT, and 5-HIAA were measured by high pressure liquid chromatography coupled to electrochemical detection (HPLC-EC) using a Phenomenex Luna 5 μm, C18, 250 mm × 4.60 mm column (Phenomenex, Torrance, CA, USA) and LC-4C electrochemical detector with glassy carbon electrode (BAS). The working electrode was set at + 0.65 V versus an Ag/AgCl reference electrode. The mobile phase contained 0.76 M NaH2PO4·H2O, 0.5 mM EDTA, 1.2 mM 1-octane sulfonic acid, and 5% acetonitrile; pH was adjusted to 3.0. Catecholamine quantification was referred to total protein content, measured using the NanoDrop 1000 Spectrophotometer (Thermo Scientific).

### cFos immunohistochemistry

Animals from the second cohort of behavioral testing were randomly selected (7 SAL-SAL, 6 SAL-VPA, 5 VPA-SAL, and 6 VPA-VPA). Two weeks after the last behavioral test, mice were deeply anesthetized with ketamine/xilacine and transcardially perfused with 4% paraformaldehide (PFA). Brains were post-fixed for 4 h in 4% PFA and criopreserved in 30% sucrose. 0.035-mm thick coronal sections were prepared on a cryostat (Leica, Wetzlar, Germany), and cFos immunohistochemistry was performed as previously described [29]. We used the primary antibody rabbit anti-c-Fos (1:1000 in blocking solution; EMD Millipore, Burlington, MA, USA), the secondary antibody biotin-SP-conjugated donkey anti-rabbit (Jackson ImmunoResearch, West Grove, PA, USA) and the ABC kit (Vector Laboratories, Burlingame, CA, USA). Sections were then stained with cresyl violet (5 mg/ml in 0.6% acetic acid). Positive cells in the layer 2 of the piriform cortex were counted on × 400 magnification in a light-field microscope (CX31: Olympus, Buenos Aires, Argentina) by a researcher (N.S.) blinded to treatments. Total c-Fos-positive nuclei are presented normalized by the volume of the layer 2. To this end, each counted section was photographed under × 40 magnification using a digital camera (Infinity 1; Lumera Corporation, Ottawa, ON, Canada) attached to the microscope. The volume of layer 2 in each image was determined using ImageJ [30]: the area in pixels was transformed to square millimeters and multiplied by the thickness of the section (0.035 mm). The limit between the anterior and posterior parts of the piriform cortex was determined with the aid of the mouse brain atlas [31] and set to the bregma position when the posterior branch of the anterior commissure is visible (AP − 0.26 mm).

### Statistical analyses

Sample sizes were estimated based on similar, previously conducted studies. No statistical methods were used to predetermine sample size. Group comparisons were done using unpaired Student’s *t* test, or two- or three-way ANOVAs for normally distributed data (confirmed by the D’Agostino & Pearson omnibus normality test), with litter as a nested factor. The statistical designs and outcomes are outlined in Additional file 1: Tables S1-S4. Whenever appropriate, Tukey’s multiple comparisons test was used for post hoc comparisons. All tests were performed with the Statistica software (version 8, StatSoft Inc., Tulsa, OK, USA), and statistical significance was assumed where *p* < 0.05. We only used statistical methods to correct for multiple testing in the PET studies. Animal exclusions in behavioral tests are specified in each behavioral test description.

## Results

### Social stimulation between PD21 and PD60 reverts the sociability alterations observed after prenatal exposure to VPA

**Fig. 1:**
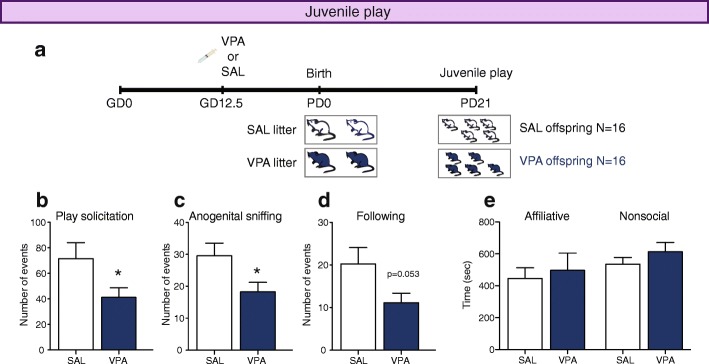
VPA-exposed animals show less social behaviors at weaning (PD21). **a** Experimental design. Pregnant dams were injected with 600 mg/kg VPA or saline on gestational day 12.5, and their offspring tested at PD21. **b**–**e** Analysis of juvenile play at weaning in VPA and SAL pairs. **b** Number of play solicitations. **c** Number of anogenital sniffs. **d** Number of follows. **e** Time spent in affiliative behaviors (sitting side-by-side, allogrooming or other) or non-social behaviors (exploring, self-grooming or sitting alone). Unpaired Student’s *t* test, * *p* < 0.05

To evaluate if social deficiencies were already present in VPA animals at weaning, we evaluated juvenile play at PD21 (Fig. 1a). VPA mice solicited play less frequently (unpaired Student’s *t* test, *t*_30_ = 2.070, *p* = 0.047; Fig. 1b), displayed less anogenital sniffing (unpaired Student’s *t* test, *t*_30_ = 2.281, *p* = 0.047; Fig. 1c) and showed a tendency to follow less their partners (unpaired Student’s *t* test with Welch’s correction, *t*_24_ = 2.035, *p* = 0.053; Fig. 1d). SAL and VPA animals did not show differences in the time spent performing affiliative behaviors (unpaired Student’s *t* test, *t*_30_ = 0.399, *p* = 0.692; Fig. 1e, left) or non-social behaviors (unpaired Student’s *t* test, *t*_30_ = 1.073, *p* = 0.292; Fig. 1e, right). These results show that VPA-exposed animals already show alterations in social behaviors at weaning.

**Fig. 2:**
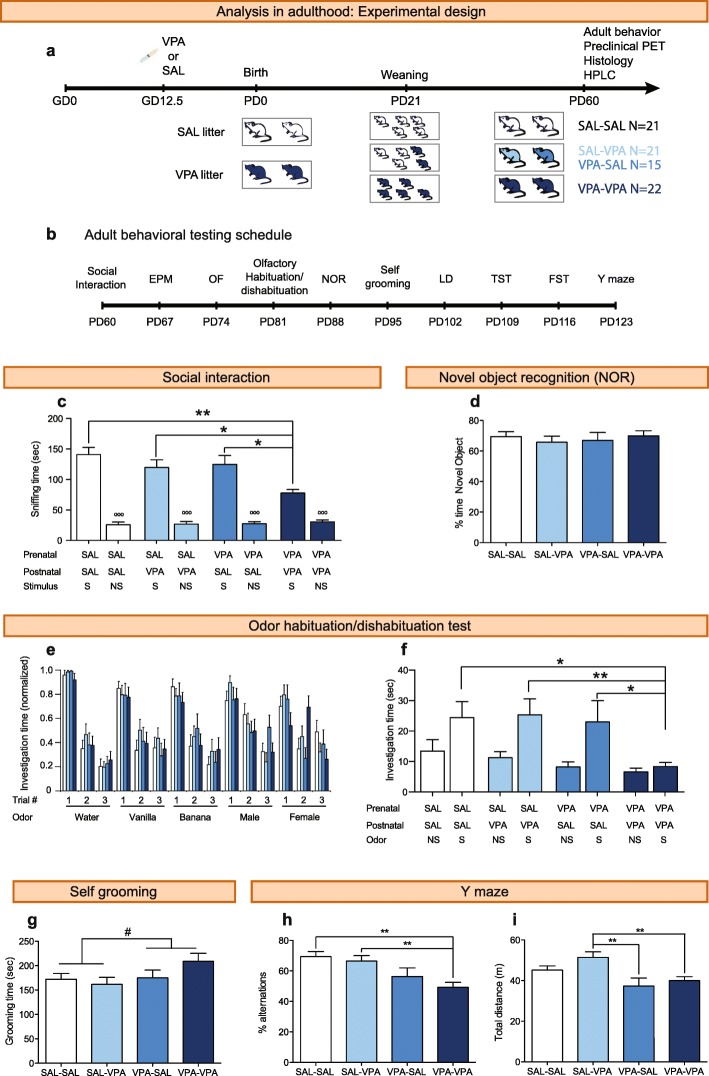
Peers can rescue the reduction in sociability observed in mice prenatally exposed to VPA, but not the increase in repetitive patterns of behavior. **a** Experimental design. Pregnant dams were injected with 600 mg/kg VPA or saline on gestational day 12.5. Offspring exposed to VPA (dark blue mice on PD21) and SAL (white mice on PD21) were weaned on PD21 in three type of cages. Due to interaction in the home cage until PD60, four experimental groups were generated: VPA animals weaned with other VPA animals (VPA-VPA group; dark blue); VPA animals weaned with saline mice (VPA-SAL group; blue); saline animals weaned with VPA animals (SAL-VPA group; light blue); and saline animals weaned with other saline animals (SAL-SAL group; white). **b** Adult behavioral analysis. Starting at PD60, all animals were subjected to a battery of behavioral tests performed in the order depicted. Tests were separated by 1-week intervals to reduce any inter-test effect. EPM, elevated plus maze; FST, forced swimming test; LD, light-dark box; NOR, novel object recognition; OF, open field; TST, tail suspension test. **c** Time spent sniffing the social (S) or non-social (NS) stimulus in the social interaction test. **d** Percentage of time exploring a novel object in the NOR test. **e**, **f** Odor habituation/dishabituation test: (E) Normalized time spent investigating each odor presentation, during three consecutive trials; (F) Time spent investigating the first presentation of non-social (NS) or social (S) odors. **g** Self-grooming test. **h** Spontaneous alternations in the Y maze. **i** Total distance traveled in the Y maze. Paired Student’s *t* test, °°°*p* < 0.001. Two-way ANOVA prenatal factor effect, ^#^*p* < 0.05. Tukey’s post hoc test, *p < 0.05, **p < 0.01, ***p < 0.001. Graphs indicate means + s.e.m. Detailed statistical information is available in Additional file 1: Table S1

To assess the effects of social stimulation on VPA-exposed animals, we weaned VPA animals either with other VPA mice (VPA-VPA animals) or with SAL mice (VPA-SAL animals) (Fig. 2a) and evaluated their adult behavior along a battery of behavioral tests (Fig. 2b). At PD60, these experimental groups were evaluated in the social interaction test and compared with SAL-SAL and SAL-VPA mice. A two-way ANOVA showed a prenatal treatment effect (*F*_1, 75_ = 6.656, *p* = 0.012) and a postnatal treatment effect (*F*_1, 75_ = 9.099, *p* = 0.003) in the time spent sniffing the stimulus mouse, with no interaction between factors (*F*_1, 75_ = 1.315, *p* = 0.255). VPA-VPA animals spent less time interacting with the social stimulus than any other group (Fig. 2c). These results confirm previous reports showing an effect of prenatal VPA exposure on adult sociability [16]. In addition, the observation that VPA-SAL animals spent more time in social interaction that VPA-VPA mice, and that their interaction time was similar to that observed in the control groups, indicate that social interactions between PD21 and PD60 can rescue alterations on social behavior due to prenatal exposure to VPA.

We were able to rule out neophobia as the cause of the decrease in social interaction since (a) all groups showed similar investigation times of the novel cylinders in the habituation phase of the social interaction test (Additional file 1: Figure S1a); (b) all groups spent a similar amount of time exploring the two identical objects in the training session of the novel object recognition test (Additional file 1: Figure S1b); and (c) all groups responded similarly to a novel object, spending around 65–70% of the total time exploring it during the testing session (two-way ANOVA, prenatal treatment: *F*_1, 74_ = 0.040, *p* = 0.842; postnatal treatment: *F*_1, 74_ = 0.009, *p* = 0.926; interaction: *F*_1, 74_ = 0.730, *p* = 0.396; Fig. 2d).

VPA-VPA mice could have alterations in olfactory function, which would likely result in altered responses to novel social stimuli. To test this, we performed the olfactory habituation/dishabituation test. We found no differences between groups in the habituation to non-social odors or male urine, with all animals showing significant habituation (Fig. 2e and Additional file 1: Table S1). However, we found a significant interaction between trial and postnatal treatment for the female odor (Three-way repeated-measure ANOVA, *F*_2, 134_ = 4.094, *p* = 0.019), with VPA-VPA mice only showing habituation to this odor in the third trial. The amount of time spent investigating novel odors can be an indicator of arousal/motivation, so we pooled all novel (trial 1) odor investigation durations across non-social (vanilla and banana) and social (male and female) odors (Fig. 2f). All groups show similar levels of investigation of non-social odors (two-way ANOVA, prenatal treatment: *F*_1, 66_ = 3.40, *p* = 0.070; postnatal treatment: *F*_1, 66_ = 0.505, *p* = 0.480; interaction: *F*_1, 66_ = 0.014, *p* = 0.906). We found a significant interaction between prenatal and postnatal treatments in the time investigating the first presentation of social odors (*F*_1, 66_ = 4.141, *p* = 0.046), with VPA-VPA mice investigating the stick less time than the other three groups (Fig. 2f). These results show that the behavioral phenotype of VPA-VPA mice is specific to social odors.

### Prenatal exposure to VPA results in repetitive patterns of behavior, which are not rescued by early social stimulation

Repetitive, stereotyped behaviors are a common behavioral phenotype observed in ASD patients. To evaluate if VPA-exposed mice exhibit repetitive or stereotyped patterns of behavior, we performed two behavioral tests: the self-grooming and the Y maze tests. We found an effect of prenatal treatment on the time spent grooming (*F*_1, 73_ = 4.215, *p* = 0.044), with VPA-exposed mice spending significantly more time in self-grooming than the offspring of SAL-injected dams (Fig. 2g). In addition, we found an effect of prenatal treatment on the percentage of alternations in the Y maze (*F*_1, 74_ = 15.501, *p* < 0.001). Post hoc analysis revealed that VPA-VPA animals alternate less than SAL-SAL or SAL-VPA animals, while VPA-SAL mice showed an intermediate performance (Fig. 2h). We also observed an effect of prenatal treatment on the total distance traveled in the Y maze (*F*_1, 74_ = 13.582, *p* < 0.001], with animals prenatally exposed to VPA showing less exploratory behavior (Fig. 2i).

These data show that the increment in repetitive behaviors observed in VPA-exposed mice is not reversed, but only partially ameliorated, by early social stimulation. Although we cannot rule it out, the similar results obtained in the self-grooming and Y maze tests suggest that they are not be affected by previous testing.

### Prenatal exposure to VPA results in reduced exploration and increased depression-related behaviors in adult mice

**Fig. 3:**
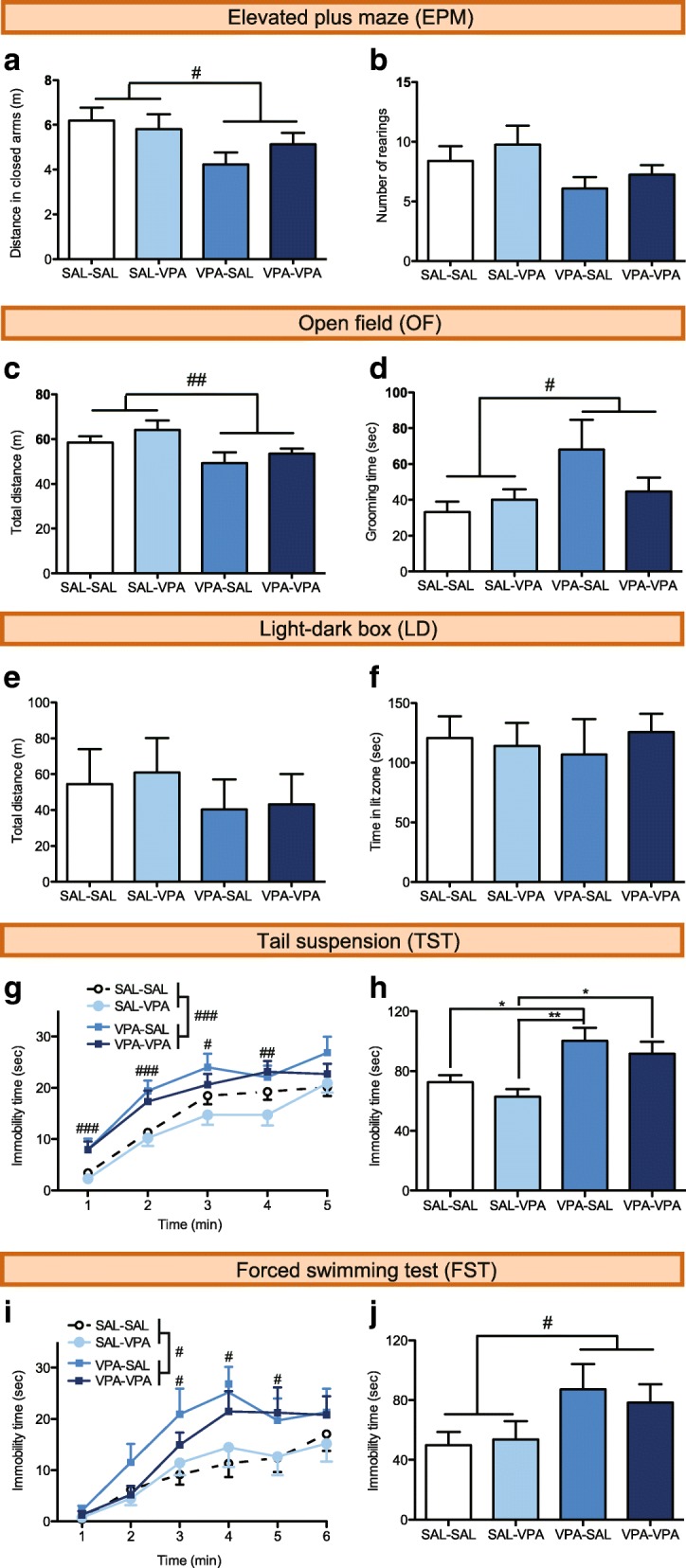
Mice prenatally exposed to VPA show reduced exploration and increased levels of depression-related behavior. **a**, **b** Distance traveled in the closed arms (**a**) and number of rearings (**b**) in the EPM. **c**, **d** Total distance traveled (**c**) and time spent grooming (**d**) in the OF. **e**, **f** Total distance traveled (**e**) and time spent (**f**) in the lit compartment in the LD box. **g**, **h** Time spent immobile in 1-min bins (**g**) and accumulated time (**h**) in the TST. **i**, **j** Time spent immobile in 1-min bins (**i**) and cumulative time between 3 and 6 min (**j**) in the FST. Two-way or repeated measures ANOVA prenatal effect, ^#^*p* < 0.05, ^##^*p* < 0.01, ^###^*p* < 0.001. Tukey’s post hoc test, *p < 0.05, **p < 0.01. Graphs indicate means + s.e.m. Detailed statistical information is available in Additional file 1: Table S2

Anxiety shows a high comorbidity with ASD [32]. To assess anxiety-related behavior, we performed the EPM, the OF, and the LD tests in the four experimental groups. We did not find evidence of anxiety-related behavior in the EPM, as all animals spent a similar amount of time in open and closed arms (Additional file 1: Table S2). However, VPA-exposed mice explored less the maze, walking less in the closed arms (two-way ANOVA prenatal effect: *F*_1, 75_ = 4.936, *p* = 0.029; Fig. 3a), and showing a tendency to perform less rearings (two-way ANOVA prenatal effect: *F*_1, 75_ = 3.835, *p* = 0.054; Fig. 3b). In addition, we observed an effect of postnatal treatment on the time spent in the center of the maze (*F*_1, 75_ = 7.079, *p* = 0.009), a zone were animals can assess risk, suggesting that early social stimulation can generate changes in the risk assessment strategy independently of the prenatal treatment. Finally, we did not observe differences between groups with regard to the number of head dippings and protected head dippings, or grooming time (Additional file 1: Table S2).

Again, we did not find evidence of anxiety-related behavior in the OF, as all animals spent a similar amount of time in the center of the field (Additional file 1: Table S2). However, animals exposed to VPA walk less (two-way ANOVA prenatal effect: *F*_1, 74_ = 7.911, *p* = 0.006; Fig. 3c) and spend more time in grooming (two-way ANOVA prenatal effect: *F*_1, 74_ = 4.872, *p* = 0.030; Fig. 3d). This last result goes in line with the evidence observed in the self-grooming test for repetitive patterns of behavior. We also observed prenatal (*F*_1, 74_ = 12.319, *p* < 0.001) and postnatal effects (*F*_1, 74_ = 4.690, *p* = 0.034) on the number of rearings in the OF, with rescued mice performing less vertical explorations than SAL animals.

In agreement with the previous results, we did not observe differences between the experimental groups in the LD test. Animals walked similar distances in the lit compartment (Fig. 3e) and spent a similar amount of time in the lit compartment (Fig. 3f) (Additional file 1: Table S2).

We also evaluated immobility in the TST and FST, a variable that is affected by the treatment with antidepressant drugs and has been interpreted as “behavioral despair”. For the TST, a three-way ANOVA with repeated measures showed only a significant effect of prenatal treatment (*F*_1, 74_ = 17.713, *p* < 0.001; Fig. 3g), with VPA-exposed animals spending more time immobile (Fig. 3g, h). Similarly, a three-way ANOVA with repeated measures showed a significant effect of prenatal treatment on the time spent immobile in the FST (*F*_1, 75_ = 6.638, *p* = 0.012; Fig. 3i) and a prenatal effect for the cumulative immobility time between 3 and 6 min (two-way ANOVA, *F*_1, 75_ = 5.981, *p* = 0.017; Fig. 3j), with VPA-exposed animals spending more time immobile.

These results collectively show that prenatal VPA exposure results in animals with reduced exploration and increased depression-related behaviors but does not impact anxiety-related behaviors. We did not observe any effects of the postnatal treatment.

### A preclinical PET analysis shows that prenatal VPA exposure results in altered patterns of brain glucose metabolism that are reversed by early social stimulation

**Fig. 4:**
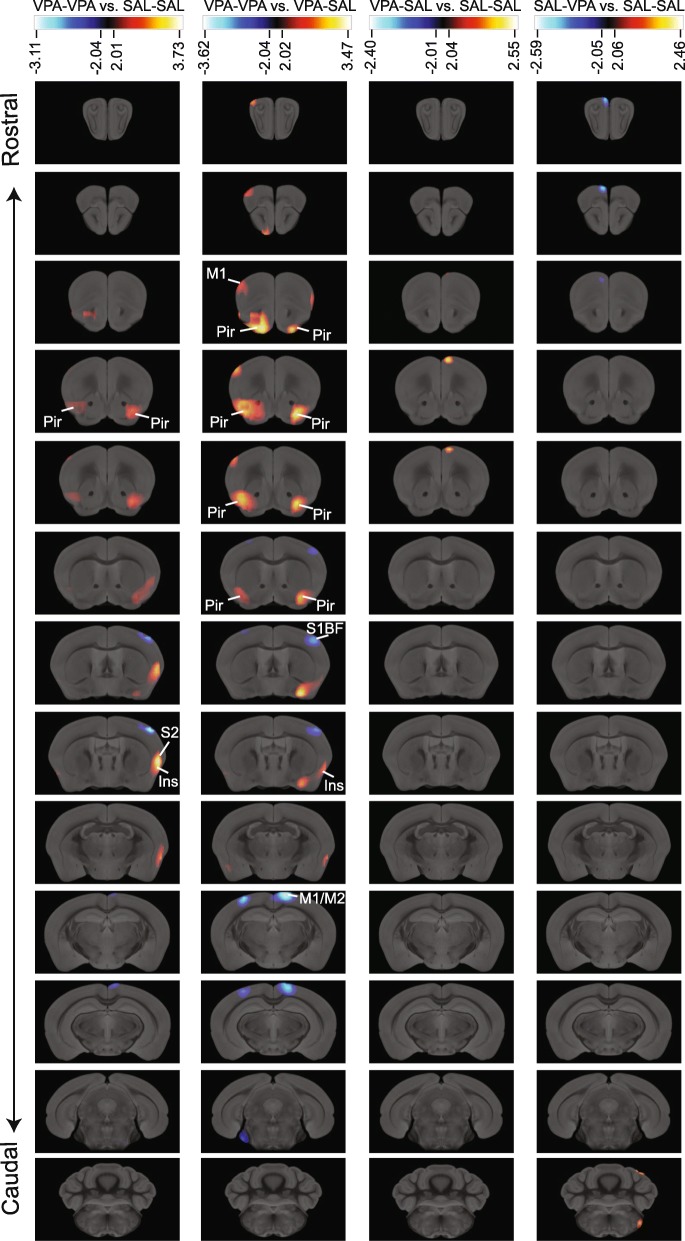
Brain areas showing significant changes in glucose metabolism induced by prenatal and postnatal treatments. Rostral-caudal coronal sections (lines) showing the comparison between the different experimental groups (columns). Significant decreases are shown blue and increases red-yellow, *t* values for *p* < 0.05 are specified for each comparison. Ins, insular cortex; M1, primary motor cortex; M2, secondary motor cortex; Pir, piriform cortex; S1BF, primary somatosensory cortex, barrel field; S2, secondary somatosensory cortex

To gain insight into the physiological changes that accompany the behavioral alterations observed after prenatal exposure to VPA and early social stimulation, we injected animals with [18F]-FDG and evaluated basal glucose metabolism 30 min after injection. We found evidence of increased glucose metabolism in the piriform cortex (Pir) of VPA-VPA animals when compared to either SAL-SAL or VPA-SAL mice (Fig. 4). This increase was bilateral and circumscribed to the anterior part of the Pir, and it was more significative when VPA-VPA animals were compared to rescued animals. We also observed increased glucose metabolism in the frontal motor cortex (M1) and reduced metabolism in the caudal part of the motor cortex (M1/M2) of VPA-VPA animals when compared with VPA-SAL mice. In addition, we observed increased glucose metabolism in the insular cortex (Ins) in VPA-VPA mice when compared with either SAL-SAL or VPA-SAL mice. Finally, we observed reduced glucose metabolism in the primary somatosensory cortex, barrel field (S1BF) in VPA-VPA mice.

Interestingly, VPA-SAL animals showed patterns of glucose metabolism more similar to SAL-SAL animals than VPA-VPA mice, suggesting that early social stimulation can revert the brain alterations caused by prenatal VPA exposure.

### c-Fos immunoreactivity and neurotransmitters alterations in the piriform cortex of VPA-VPA mice are alleviated by early social stimulation

**Fig. 5:**
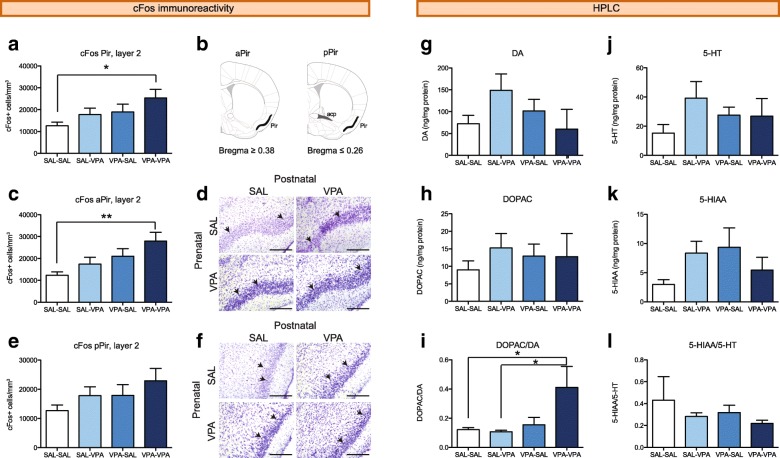
VPA-VPA mice show increased c-Fos immunoreactivity and dopaminergic turnover in the piriform cortex. **a**–**f** Density of c-Fos-positive nuclei was measured in the whole Pir (**a**). Schemes in **b** show the posterior limit of the aPir and the anterior limit of the pPir, where the posterior part of the anterior commissure (acp, dark gray) is visible. Data is also presented discriminating between the anterior part of the Pir (**c**, aPir) and the posterior part of the Pir (**e**, pPir). *n* = 5–7 mice/group. Representative images of the aPir (**d**) and pPir (**f**) for the four experimental groups are shown. c-Fos-positive nuclei (black, arrows show examples) in Nissl stained slices. Bars, 200 μm. **g**–**l** Monoamine levels in the Pir were measured by HPLC-ED. **g** DA, **h** DOPAC, **i** DA turnover (DOPAC/DA), **j** 5-HT, **k** 5-HIAA, and **l** 5-HT turnover (5-HIAA/5-HT). *n* = 4–5 mice/group. Two-way ANOVA followed by Tukey’s post hoc tests, *p < 0.05, **p < 0.01. Graphs indicate means + s.e.m. Detailed statistical information is available in Additional file 1: Table S3

The increase in glucose metabolism in the Pir of VPA-VPA mice observed with the preclinical PET could be due to increased cellular activity. c-Fos is an immediate early gene whose expression is stimulated by neuronal depolarization and has been previously used to map changes in neuronal activity due to altered excitation/inhibition balance [33, 34]. We counted the number of c-Fos-positive nuclei in the layer 2 of the Pir and found it increased in VPA-VPA mice compared to SAL-SAL mice (Fig. 5a). To further characterize this increment, we analyzed the anterior and posterior parts of the Pir (Fig. 5b), and found it particularly significant in the aPir (Fig. 5c, d, Additional file 1: Figure S2a-d). Interestingly, both SAL-VPA and VPA-SAL mice exhibit an intermediate phenotype. Differences in the density of c-Fos-positive cells in the pPir were not statistically significant (Fig. 5e, f, and Additional file 1: Figure S2e–h). We found no c-Fos-positive cells in the layer 1 of the Pir, and only few c-Fos-positive cells in the layer 3 (Additional file 1: Figure S2), where we found no differences between groups (Additional file 1: Table S3).

The Pir is highly innervated by serotoninergic neurons of the raphe nucleus [35] and midbrain dopaminergic neurons [36], neurotransmitters that can modulate the activity of principal cells and interneurons [37, 38]. To evaluate dopaminergic and serotoninergic function in the Pir, we measured the levels of these neurotransmitters and their metabolites by HPLC-ED. We found no differences between groups neither in the levels of dopamine (DA; Fig. 5g) or its metabolite DOPAC (Fig. 5h), serotonin (5-HT; Fig. 5j) or its metabolite 5-HIAA (Fig. 5k) nor the 5-HIAA/5-HT ratio (Fig. 5l). However, we found an increased dopaminergic turnover in the Pir of VPA-VPA mice when compared with mice prenatally exposed to SAL (Fig. 5i).

These results show that VPA-VPA mice have altered neuronal function in the Pir, and early social stimulation at least partially reverts these alterations.

## Discussion

Taken together, our results show that early social stimulation can revert the decrease in sociability observed in VPA-exposed mice, while other behaviors are not affected by this postnatal treatment. In addition, preclinical PET analysis identified specific brain regions whose metabolism is altered by prenatal VPA treatment. Interestingly, most of these regions regain the control levels when VPA animals are reared with SAL mice. Among these brain structures, the piriform cortex (Pir) shows a bilaterally increased metabolism in VPA-VPA animals when compared with SAL-SAL or VPA-SAL mice. We also observed that VPA-VPA mice have more cFos-positive cells in the aPir and increased dopamine turnover in the Pir.

Performing a battery of behavioral tests and measuring a significant number of physiological variables leads to the problem of multiple testing when performing statistical analyses of the results. We reasoned that methods that provide more strict *p* values (e.g., Bonferroni correction or FDR) could contribute to avoiding false positives, but because of the extensive nature of our analysis, they could also lead to false negatives. VPA treatment, social enrichment, and the use of an outbred strain, all contribute to biological variability, making it difficult to detect meaningful differences. As an alternative, we have employed different strategies in order to minimize the effect of multiple testing on our results. On one hand, we used independent cohorts for juvenile play, PET studies (two cohorts), and HPLC analysis. In addition, adult behavioral testing was performed in three different cohorts, and each behavioral domain was evaluated in more than one test and variable. Moreover, animals employed for c-Fos expression analysis were randomly selected from a behavioral cohort, and a FDR approach was used in PET studies. All these approaches contribute to reducing type 1 error without significantly increasing type 2 error and the number of animals used for experiments.

The development, organization, and function of the social brain circuitry result from the interaction between the child and his/her social environment [11]. Reciprocal social interactions are required for the integration of social stimuli to engage brain regions involved in reward, motor control, and attention [39]. In mammals, social interactions take place first with the parents and subsequently with the peers. Altered social environments can worsen genetic or prenatal factors affecting sociability, and we reasoned that, conversely, a rich social environment could counteract prenatal factors affecting sociability. Indeed, most early behavioral interventions employed in treating ASD target social aspects of behavior (e.g., language, social interaction) [40].

We have previously shown that maternal behavior is not affected by injecting VPA to the dams at GD12.5 [41], suggesting that maternal care is not contributing to the social deficits observed at weaning (Fig. 2a–c). After weaning, animals only interact with peers in the home cage. We hypothesized that the poor social environment experienced by VPA-exposed mice reared with other VPA-exposed mice could contribute to the reduction in sociability observed in adulthood. In addition, we hypothesized that VPA-exposed animals reared with animals showing more sociability could reverse their phenotype. Our design allowed us to specifically test the role of reciprocal interactions, without the confounding effect of the mother that could emerge from earlier interventions. Moreover, the developmental processes taking place in the rodent brain from PD20-PD25 are similar to those occurring in the 2–5-year-old human brain [42], when usually ASD symptoms are detected.

We found that post-weaning social enrichment can reverse the altered sociability observed in young VPA-exposed mice, resulting in adult animals showing more social interaction that VPA-exposed animals reared with other VPA mice. Particularly, we show that VPA-VPA animals spend less time sniffing a novel mouse than any of the other groups (SAL-SAL, SAL-VPA, or VPA-SAL). The maze that we have employed in this work, originally described in [43], consists of small chambers. As a consequence, when animals are located in the lateral chambers they are mostly exploring the cylinder, rarely exploring the maze. Therefore, “sniffing” is the most reliable parameter of sociability since it involves the actual investigation of the social stimulus or the exploration of the control tube. However, it also implies a limitation because we can only provide one measure of social interaction. To evaluate the specificity of this effect, we analyzed other behaviors that are altered by prenatal VPA-exposure. We found that the VPA effects on repetitive behaviors, exploration, and depression-related behavior are not reversed by early social enrichment (Figs. 3 and 4). These results suggest that the previous reported effects of enriched environments on these behaviors [14, 44, 45] are due to the opportunity to explore a richer physical environment and not to the social stimulation available in such environments.

Alterations in juvenile play are observable at PD21 in mice prenatally exposed to VPA. Our results suggest that there is an additional critical period starting around PD21, when the interactions with peers can set the level of sociability in mice. Although we cannot claim a specific critical period of development for this effect, previous work suggests adolescence (approximately from PD21 to PD42) as a critical period for the establishment of social behaviors [reviewed in [46]]. Future work could narrow this critical period of postnatal development and identify the mechanisms through which they act. Although VPA and SAL animals show different levels of sociability at PD21 and the only variable we modified was the composition of the cages from PD21 to PD60, we did not specifically evaluate the levels of social stimulation on VPA-exposed animals in VPA-VPA and VPA-SAL groups. Therefore, we cannot rule out the contribution of additional unknown parameters (e.g., microbiota in the fecal matter [47]) that might affect the development and consolidation of sociability.

Here, we report the effects of VPA and early social stimulation on male offspring. We circumscribed our analysis to males since we did not observe any effects of prenatal VPA on social interactions in female offspring compared with the controls (unpublished results). This is consistent with the reports in CD1 mice [48], Sprague-Dawley rats [20], and Wistar rats [17]. This differential impact of VPA on male and female offspring adds face validity to this ASD model, as this disorder is four times more common in males than in females [49]. However, the prevalence of autism in children prenatally exposed to VPA is characterized by a 1:1 male to female ratio [50], and in the VPA model, there is an even effect on both sexes in other ASD-related phenotypes such as anxiety-related behaviors and repetitive behaviors [17, 48]. Moreover, female VPA offspring exhibit physiological and cellular changes that are consistent with the alterations observed in ASD children [17, 41, 51]. However, to date, sociability defects in female mice as a result of VPA exposure have not been found. The effects of social enrichment on females prenatally exposed to VPA needs to be further analyzed, as it can contribute to the treatment of girls diagnosed with ASD.

Most of our knowledge on brain circuit abnormalities in ASD individuals comes from functional MRI studies [5, 52]. However, these reports show numerous inconsistencies on the brain areas involved. We reasoned that our rescued group of animals would be a valuable tool to validate alterations observed in the VPA model. Using [18F]-FDG preclinical PET, we found that brain activity of VPA-SAL animals is more similar to SAL-SAL brains than VPA-VPA brains, as we found alterations in metabolism in the VPA-VPA brain that are not present in rescued animals. These structures appear then as candidate components of the social brain circuitry of the mouse. Interestingly, our results show differences in glucose metabolism in the resting state, similar to studies describing altered connectivity in people with ASD [5].

The circuitry that regulates social behavior involves many brain structures. In rodents, these structures are mainly components of the olfactory system, such as the anterior olfactory nucleus (AON) and the Pir. Indeed, the Pir controls olfactory perception and is activated by social stimuli [53], and during the discrimination between familiar and non-familiar social stimuli [54, 55]. Moreover, the oxytocin receptor-expressing neurons in the Pir mediate odor-driven social learning [56]. In line with this, we found a bilateral hyperactivation of the Pir in adult VPA-VPA mice, when compared to either control or rescued mice (Fig. 4). Moreover, we found increased cFos immunoreactivity in the layer 2 of the aPir (Fig. 5c), suggesting increased basal neuronal activity in VPA-VPA mice. Although we did not identify the type of neurons expressing c-Fos, the fact that they are localized for the most part in the layer 2 together with their morphology suggest that they are mostly glutamatergic principal neurons.

The Pir projects to the amygdala, basal ganglia, and hippocampus, areas implicated in behavioral output, with the aPir particularly projecting to the anterior part of the amygdala and the mediodorsal thalamus [57]. Alterations in the aPir function could then result in alterations in the circuitry engaged during social interactions. The Pir shows increased cFos immunoreactivity after exposure to a juvenile conspecific, but it is reduced after a neutral odor [55]. The increased basal neuronal activity observed in VPA-VPA mice could then preclude the circuitry to respond normally to a social stimulus, leaving the response to non-social odors unaffected (Fig. 2e, f).

In addition, we found increased dopaminergic turnover in the Pir of VPA-VPA mice (Fig. 5i). Cells in the Pir receive input from midbrain dopaminergic neurons [36], express dopamine receptors [58], and exhibit dose-dependent responses to dopamine [59]. Rodent dopaminergic projections and dopamine receptor expression in the brain undergo profound changes during the juvenile (PD21-PD28) and adolescent (PD34-PD49) stages [60, 61]. Social enrichment could modify a VPA-induced aberrant pattern of maturation of dopaminergic innervations, restoring normal dopaminergic function. Coactivation of dopamine D1 and D2 receptors results in reduced social interaction [62], suggesting a possible mechanism by which increased dopaminergic function in the Pir could result in diminished sociability in VPA-VPA mice.

In summary, VPA-VPA mice show reduced sociability and increases in glucose metabolism, neuronal activity, and dopaminergic turnover. In turn, VPA-SAL mice show no significant differences in any of these parameters when compared with SAL-SAL mice, displaying a phenotype that was indistinguishable from that of control animals. In addition, VPA-SAL sociability levels are significantly higher and glucose metabolism in the Pir is significantly lower when compared with VPA-VPA mice. These differences are partially explained by the reduction in neuronal activity and dopaminergic turnover observed in VPA-SAL mice, but additional neuronal changes in the Pir may be involved, as it is suggested by the larger differences observed in the PET between both VPA-exposed groups than in the VPA-VPA vs SAL-SAL comparison.

Our preclinical PET results also show alterations in glucose metabolism in other brain structures, the motor cortex (M1/M2), the somatosensory cortex (S2 and S1BF) and the insular cortex. We need to further study these structures to elucidate if they play a role in modulating the behaviors altered in animals prenatally exposed to VPA. It was recently shown that maternal immune activation (MIA) results in cortical patches of reduced cellular activity (cFos-positive cells) in the primary somatosensory cortex (S1) and secondary motor cortex (M2) [34]. Interestingly, these patches were often present unilaterally, similar to the pattern that we observed (Fig. 4). MIA offspring show behavioral alterations comparable to those observed in VPA-exposed mice, and the specific manipulation of neuronal activity on the S1 can modulate social interaction and repetitive behaviors in MIA offspring [34]. These results demonstrate a role of the S1 in modulating these behaviors and suggest that the reduction in glucose metabolism observed unilaterally in S1 and M2 in VPA-VPA mice could also contribute to the abnormal behavior of these animals.

Our unbiased analysis of brain activity in a mouse model of ASD adds to previous fMRI studies on brain connectivity and neuronal activity in the acallosal and socially impaired BTBR strain [63, 64]. BTBR mice showed reduced basal neuronal activity and metabolism in the somatosensory and piriform cortex, when compared with C57BL/6J mice. In addition, brain activity mapping was previously performed in a mouse model of Rett syndrome (the Mecp2 null mice), using a histological technique (Fos labeling) [33]. Interestingly, that analysis reports reduced neuronal activity in the Pir, somatosensory, and motor cortices. As Mecp2 null mice [65] and BTBR mice [66] show deficits in social behavior, our results add to those reports in suggesting that these structures could be central to its regulation.

## Conclusions

In conclusion, this study demonstrates that social enrichment after weaning can reverse the detrimental effects of prenatal VPA exposure on sociability. Our model of rescued mice can then be valuable to test whether molecular and cellular alterations can be reversed in the VPA model when social deficits are reversed. As other ASD-related behaviors are not affected by the rearing protocol, this model can also be used to disentangle the circuits that are engaged in social and repetitive behaviors. Finally, our results highlight the relevance of studying the role of the Pir in modulating social behavior.

## Additional file

Additional file 1: **Table S1.** Summary of ANOVA results for the behavioral analyses conducted on adult offspring in the four experimental groups (data corresponding to main Fig. 2). **Table S2.** Summary of ANOVA results for the behavioral analyses conducted on adult offspring in the four experimental groups (data corresponding to main Fig. 3). **Table S3.** Summary of ANOVA results for the histological analyses and HPLC conducted on adult offspring in the four experimental groups (data corresponding to main Fig. 5). **Figure S1.** Animals show normal responses to novel objects. **Figure S2.** Animals show similar numbers of cFos-positive cells in the layer 3 of the piriform cortex. **Figure S3.** Representative images of sections processed for c-Fos immunoreativity and Nissl staining (DOCX 729 kb)

## Abbreviations

ASD: Autism spectrum disorder
EPM: Elevated plus maze test
FST: Forced swimming test
LD: Light-dark box test
NOR: Novel object recognition test
OF: Open field test
Pir: Piriform cortex
TST: Tail suspension test
VPA: Valproic acid
