# Supplementary material for: Sociability deficits after prenatal exposure to valproic acid are rescued by early social enrichment
Source: Mol Autism. 2018 Jun 14;9:36. doi: 10.1186/s13229-018-0221-9 (PMC6001054; doi:10.1186/s13229-018-0221-9)
Supplement: Supplementary file 1 — Table S1. Summary of ANOVA results for the behavioral analyses conducted on adult offspring in the four experimental groups (data corresponding to main Fig. 2). Table S2. Summary of ANOVA results for the behavioral analyses conducted on adult offspring in the four experimental groups (data corresponding to main Fig. 3). Table S3. Summary of ANOVA results for the histological analyses and HPLC conducted on adult offspring in the four experimental groups (data corresponding to main Fig. 5). Figure S1. Animals show normal responses to novel objects. Figure S2. Animals show similar numbers of cFos-positive cells in the layer 3 of the piriform cortex. Figure S3. Representative images of sections processed for c-Fos immunoreativity and Nissl staining (DOCX 729 kb) [file 13229_2018_221_MOESM1_ESM.docx]

**SUPPLEMENTARY INFORMATION**

**Sociability deficits after prenatal exposure to Valproic Acid are rescued by early social enrichment**

Marcos Campolongo^1,2^, Nadia Kazlauskas^1,2^, German Falasco^3^, Leandro Urrutia^3^, Natalí Salgueiro^1,2^, Christian Höcht^4^, Amaicha Mara Depino^1,2^

^1^ Universidad de Buenos Aires, Facultad de Ciencias Exactas y Naturales, Departamento de Fisiología, Biología Molecular y Celular, Buenos Aires, Argentina

^2^ CONICET-Universidad de Buenos Aires, Instituto de Fisiología, Biología Molecular y Neurociencias (IFIBYNE), Buenos Aires, Argentina.

^3^ FLENI, Centro de Imágenes Moleculares, Laboratorio de Imágenes Preclínicas, Buenos Aires, Argentina.

^4^ Universidad de Buenos Aires, Facultad de Farmacia y Bioquímica, Cátedra de Farmacología, Buenos Aires, Argentina.

**Corresponding author:** Amaicha Mara Depino, PhD. IFIByNE, UBA-CONICET. Int. Guiraldes 2160, Ciudad Universitaria, Pabellon 2, Ciudad de Buenos Aires, Argentina. Tel: +5411-4576-3386 extension 108. Email: [adepino@conicet.gov.ar](mailto:adepino@conicet.gov.ar)

**SUPPLEMENTARY TABLES**

| **Test** | **Dependent measures** | **ANOVA** | **Effects** | **DF** | ***F*-Value** | ***p*-Value** |
| --- | --- | --- | --- | --- | --- | --- |
| **Social interaction** | Time spent sniffing stimulus mouse (sec) | 2 × 2 (Prenatal × Postnatal) | Prenatal | (1, 75) | 6.656 | **0.012** |
|  |  |  | Postnatal | (1, 75) | 9.099 | **0.003** |
|  |  |  | Prenatal × Postnatal | (1, 75) | 1.315 | 0.255 |
| **Novel object recognition** | Time in Novel Object (%) | 2 × 2 (Prenatal × Postnatal) | Prenatal | (1, 74) | 0.040 | 0.842 |
|  |  |  | Postnatal | (1, 74) | 0.009 | 0.926 |
|  |  |  | Prenatal × Postnatal | (1, 74) | 0.730 | 0.396 |
| **Olfactory habituation/**  **dishabituation** | Time exploring (sec), water | 2 × 2 x 3 (Prenatal × Postnatal x Trial) | Prenatal x Trial | (2, 134) | 0.344 | 0.710 |
|  |  |  | Postnatal x Trial | (2, 134) | 0.385 | 0.681 |
|  |  |  | Prenatal × Postnatal x Trial | (2, 134) | 0.446 | 0.641 |
|  | Time exploring (sec), vanilla | 2 × 2 x 3 (Prenatal × Postnatal x Trial) | Prenatal x Trial | (2, 134) | 0.094 | 0.911 |
|  |  |  | Postnatal x Trial | (2, 134) | 0.384 | 0.682 |
|  |  |  | Prenatal × Postnatal x Trial | (2, 134) | 0.333 | 0.717 |
|  | Time exploring (sec), banana | 2 × 2 x 3 (Prenatal × Postnatal x Trial) | Prenatal x Trial | (2, 134) | 0.280 | 0.756 |
|  |  |  | Postnatal x Trial | (2, 134) | 0.812 | 0.446 |
|  |  |  | Prenatal × Postnatal x Trial | (2, 134) | 0.418 | 0.659 |
|  | Time exploring (sec), male swap | 2 × 2 x 3 (Prenatal × Postnatal x Trial) | Prenatal x Trial | (2, 134) | 1.858 | 0.160 |
|  |  |  | Postnatal x Trial | (2, 134) | 1.354 | 0.262 |
|  |  |  | Prenatal × Postnatal x Trial | (2, 134) | 0.436 | 0.647 |
|  | Time exploring (sec), female swap | 2 × 2 x 3 (Prenatal × Postnatal x Trial) | Prenatal x Trial | (2, 134) | 0.866 | 0.710 |
|  |  |  | Postnatal x Trial | (2, 134) | 4.094 | **0.019** |
|  |  |  | Prenatal × Postnatal x Trial | (2, 134) | 2.230 | 0.112 |
|  | Investigation time (sec), non-social odors | 2 × 2 (Prenatal × Postnatal) | Prenatal | (1, 66) | 0.340 | 0.070 |
|  |  |  | Postnatal | (1, 66) | 0.505 | 0.480 |
|  |  |  | Prenatal × Postnatal | (1, 66) | 0.014 | 0.906 |
|  | Investigation time (sec), social odors | 2 × 2 (Prenatal × Postnatal) | Prenatal | (1, 66) | 2.679 | 0.106 |
|  |  |  | Postnatal | (1, 66) | 1.296 | 0.259 |
|  |  |  | Prenatal × Postnatal | (1, 66) | 4.141 | **0.046** |
| **Self-grooming** | Grooming time (sec) | 2 × 2 (Prenatal × Postnatal) | Prenatal | (1, 73) | 4.215 | **0.044** |
|  |  |  | Postnatal | (1, 73) | 0.233 | 0.631 |
|  |  |  | Prenatal × Postnatal | (1, 73) | 3.487 | 0.066 |
| **Y maze** | Alternations (%) | 2 × 2 (Prenatal × Postnatal) | Prenatal | (1, 74) | 15.501 | **<0.001** |
|  |  |  | Postnatal | (1, 74) | 1.705 | 0.195 |
|  |  |  | Prenatal × Postnatal | (1, 74) | 0.283 | 0.596 |
|  | Total distance (m) | 2 × 2 (Prenatal × Postnatal) | Prenatal | (1, 74) | 13.582 | **<0.001** |
|  |  |  | Postnatal | (1, 74) | 2.942 | 0.090 |
|  |  |  | Prenatal × Postnatal | (1, 74) | 0.472 | 0.494 |

***Table S1.*** Summary of ANOVA results for the behavioral analyses conducted on adult offspring in the four experimental groups (data corresponding to main *Figure 2*). The table specifies the F values for each analyzed behavior, along with the corresponding degrees of freedom (DF). Significant effects (*p* < 0.05) are given in bold font. *n* = 13-22 per group.

| **Test** | **Dependent measures** | **ANOVA** | **Effects** | **DF** | ***F*-Value** | ***p*-Value** |
| --- | --- | --- | --- | --- | --- | --- |
| **Elevated plus maze** | Distance in closed arms (m) | 2 × 2 (Prenatal × Postnatal) | Prenatal | (1, 75) | 4.936 | **0.029** |
|  |  |  | Postnatal | (1, 75) | 0.189 | 0.665 |
|  |  |  | Prenatal × Postnatal | (1, 75) | 1.138 | 0.289 |
|  | Rearings (number) | 2 × 2 (Prenatal × Postnatal) | Prenatal | (1, 75) | 3.835 | 0.054 |
|  |  |  | Postnatal | (1, 75) | 1.054 | 0.308 |
|  |  |  | Prenatal × Postnatal | (1, 75) | 0.008 | 0.929 |
|  | Time in the center (sec) | 2 × 2 (Prenatal × Postnatal) | Prenatal | (1, 75) | 0.176 | 0.676 |
|  |  |  | Postnatal | (1, 75) | 7.080 | **0.009** |
|  |  |  | Prenatal × Postnatal | (1, 75) | 0.734 | 0.394 |
|  | Head dippings (number) | 2 × 2 (Prenatal × Postnatal) | Prenatal | (1, 75) | 0.224 | 0.637 |
|  |  |  | Postnatal | (1, 75) | 0.042 | 0.838 |
|  |  |  | Prenatal × Postnatal | (1, 75) | 0.190 | 0.279 |
|  | Protected head dippings (number) | 2 × 2 (Prenatal × Postnatal) | Prenatal | (1, 75) | 0.005 | 0.942 |
|  |  |  | Postnatal | (1, 75) | 0.685 | 0.411 |
|  |  |  | Prenatal × Postnatal | (1, 75) | 1.557 | 0.216 |
|  | Time in open arms (sec) | 2 × 2 (Prenatal × Postnatal) | Prenatal | (1, 75) | 2.838 | 0.096 |
|  |  |  | Postnatal | (1, 75) | 0.001 | 0.993 |
|  |  |  | Prenatal × Postnatal | (1, 75) | 0.762 | 0.385 |
|  | Time in closed arms (sec) | 2 × 2 (Prenatal × Postnatal) | Prenatal | (1, 75) | 1.133 | 0.291 |
|  |  |  | Postnatal | (1, 75) | 3.696 | 0.058 |
|  |  |  | Prenatal × Postnatal | (1, 75) | 0.008 | 0.927 |
|  | Grooming time (sec) | 2 × 2 (Prenatal × Postnatal) | Prenatal | (1, 75) | 0.533 | 0.468 |
|  |  |  | Postnatal | (1, 75) | 0.277 | 0.600 |
|  |  |  | Prenatal × Postnatal | (1, 75) | 0.200 | 0.656 |
| **Open field** | Total distance (m) | 2 × 2 (Prenatal × Postnatal) | Prenatal | (1, 74) | 7.911 | **0.006** |
|  |  |  | Postnatal | (1, 74) | 1.955 | 0.166 |
|  |  |  | Prenatal × Postnatal | (1, 74) | 0.038 | 0.847 |
|  | Grooming time (sec) | 2 × 2 (Prenatal × Postnatal) | Prenatal | (1, 74) | 4.872 | **0.030** |
|  |  |  | Postnatal | (1, 74) | 0.856 | 0.358 |
|  |  |  | Prenatal × Postnatal | (1, 74) | 2.895 | 0.093 |
|  | Rearings (number) | 2 × 2 (Prenatal × Postnatal) | Prenatal | (1, 74) | 12.319 | **<0.001** |
|  |  |  | Postnatal | (1, 74) | 4.690 | **0.034** |
|  |  |  | Prenatal × Postnatal | (1, 74) | 1.004 | 0.320 |
|  | Time in the center (sec) | 2 × 2 (Prenatal × Postnatal) | Prenatal | (1, 74) | 0.037 | 0.848 |
|  |  |  | Postnatal | (1, 74) | 2.765 | 0.101 |
|  |  |  | Prenatal × Postnatal | (1, 74) | 0.552 | 0.460 |
| **Light-dark box** | Distance walked in Lit Compartment (m) | 2 × 2 (Prenatal × Postnatal) | Prenatal | (1, 75) | 0.735 | 0.384 |
|  |  |  | Postnatal | (1, 75) | 0.061 | 0.806 |
|  |  |  | Prenatal × Postnatal | (1, 75) | 0.010 | 0.923 |
|  | Time in Lit Compartment (sec) | 2 × 2 (Prenatal × Postnatal) | Prenatal | (1, 75) | 0.003 | 0.954 |
|  |  |  | Postnatal | (1, 75) | 0.090 | 0.765 |
|  |  |  | Prenatal × Postnatal | (1, 75) | 0.400 | 0.529 |
| **Tail suspension test** | Time immobile (sec), total | 2 × 2 x 3 (Prenatal × Postnatal x Trial) | Prenatal | (1, 74) | 17.713 | **<0.0001** |
|  |  |  | Postnatal | (1, 74) | 1.851 | 0.178 |
|  |  |  | Prenatal × Postnatal | (1, 74) | 0.008 | 0.931 |
|  |  |  | Prenatal x Trial | (4, 296) | 0.677 | 0.608 |
|  |  |  | Postnatal x Trial | (4, 296) | 0.538 | 0.708 |
|  |  |  | Prenatal × Postnatal x Trial | (4, 296) | 1.607 | 0.172 |
|  | Time immobile (sec), 1st min | 2 × 2 (Prenatal × Postnatal) | Prenatal | (1, 74) | 15.110 | **0.0002** |
|  |  |  | Postnatal | (1, 74) | 0.200 | 0.656 |
|  |  |  | Prenatal × Postnatal | (1, 74) | 0.183 | 0.670 |
|  | Time immobile (sec), 2nd min | 2 × 2 (Prenatal × Postnatal) | Prenatal | (1, 74) | 17.511 | **<0.0001** |
|  |  |  | Postnatal | (1, 74) | 0.757 | 0.387 |
|  |  |  | Prenatal × Postnatal | (1, 74) | 0.074 | 0.787 |
|  | Time immobile (sec), 3rd min | 2 × 2 (Prenatal × Postnatal) | Prenatal | (1, 74) | 6.870 | **0.011** |
|  |  |  | Postnatal | (1, 74) | 3.050 | 0.085 |
|  |  |  | Prenatal × Postnatal | (1, 74) | 0.007 | 0.931 |
|  | Time immobile (sec), 4th min | 2 × 2 (Prenatal × Postnatal) | Prenatal | (1, 74) | 7.826 | **0.007** |
|  |  |  | Postnatal | (1, 74) | 0.880 | 0.351 |
|  |  |  | Prenatal × Postnatal | (1, 74) | 1.865 | 0.176 |
|  | Time immobile (sec), 5th min | 2 × 2 (Prenatal × Postnatal) | Prenatal | (1, 74) | 3.309 | 0.073 |
|  |  |  | Postnatal | (1, 74) | 0.520 | 0.473 |
|  |  |  | Prenatal × Postnatal | (1, 74) | 1.318 | 0.255 |
| **Forced swimming test** | Time immobile (sec), total | 2 × 2 x 3 (Prenatal × Postnatal x Trial) | Prenatal | (1, 75) | 6.638 | **0.012** |
|  |  |  | Postnatal | (1, 75) | 0.241 | 0.625 |
|  |  |  | Prenatal × Postnatal | (1, 75) | 0.454 | 0.503 |
|  |  |  | Prenatal x Trial | (5, 375) | 2.224 | 0.051 |
|  |  |  | Postnatal x Trial | (5, 375) | 0.532 | 0.752 |
|  |  |  | Prenatal × Postnatal x Trial | (5, 375) | 0.791 | 0.557 |
|  | Time immobile (sec), 1st min | 2 × 2 (Prenatal × Postnatal) | Prenatal | (1, 75) | 2.993 | 0.088 |
|  |  |  | Postnatal | (1, 75) | 0.087 | 0.769 |
|  |  |  | Prenatal × Postnatal | (1, 75) | 1.000 | 0.321 |
|  | Time immobile (sec), 2nd min | 2 × 2 (Prenatal × Postnatal) | Prenatal | (1, 75) | 1.475 | 0.228 |
|  |  |  | Postnatal | (1, 75) | 3.642 | 0.060 |
|  |  |  | Prenatal × Postnatal | (1, 75) | 1.309 | 0.256 |
|  | Time immobile (sec), 3rd min | 2 × 2 (Prenatal × Postnatal) | Prenatal | (1, 75) | 6.122 | **0.016** |
|  |  |  | Postnatal | (1, 75) | 0.283 | 0.596 |
|  |  |  | Prenatal × Postnatal | (1, 75) | 2.057 | 0.156 |
|  | Time immobile (sec), 4th min | 2 × 2 (Prenatal × Postnatal) | Prenatal | (1, 75) | 6.962 | **0.010** |
|  |  |  | Postnatal | (1, 75) | 0.000 | 0.995 |
|  |  |  | Prenatal × Postnatal | (1, 75) | 0.796 | 0.375 |
|  | Time immobile (sec), 5th min | 2 × 2 (Prenatal × Postnatal) | Prenatal | (1, 75) | 4.081 | **0.047** |
|  |  |  | Postnatal | (1, 75) | 0.043 | 0.837 |
|  |  |  | Prenatal × Postnatal | (1, 75) | 0.024 | 0.877 |
|  | Time immobile (sec), 6th min | 2 × 2 (Prenatal × Postnatal) | Prenatal | (1, 75) | 1.742 | 0.191 |
|  |  |  | Postnatal | (1, 75) | 0.109 | 0.742 |
|  |  |  | Prenatal × Postnatal | (1, 75) | 0.032 | 0.858 |
|  | Time immobile (sec), 3-6 min | 2 × 2 (Prenatal × Postnatal) | Prenatal | (1, 75) | 5.981 | **0.017** |
|  |  |  | Postnatal | (1, 75) | 0.025 | 0.876 |
|  |  |  | Prenatal × Postnatal | (1, 75) | 0.254 | 0.616 |

***Table S2.*** Summary of ANOVA results for the behavioral analyses conducted on adult offspring in the four experimental groups (data corresponding to main *Figure 3*). The table specifies the F values for each analyzed behavior, along with the corresponding degrees of freedom (DF). Significant effects (*p* < 0.05) are given in bold font. *n* = 15-22 per group.

| **Analysis** | **Dependent measures** | **ANOVA** | **Effects** | **DF** | ***F*-Value** | ***p*-Value** |
| --- | --- | --- | --- | --- | --- | --- |
| **cFos** | cFos-positive cell density (number/mm^3^), total layer 2 | 2 × 2 (Prenatal × Postnatal) | Prenatal | (1, 20) | 5.154 | **0.034** |
|  |  |  | Postnatal | (1, 20) | 3.575 | 0.073 |
|  |  |  | Prenatal × Postnatal | (1, 20) | 0.042 | 0.840 |
|  | cFos-positive cell density (number/mm^3^), aPir layer 2 | 2 × 2 (Prenatal × Postnatal) | Prenatal | (1, 20) | 2.554 | 0.126 |
|  |  |  | Postnatal | (1, 20) | 2.464 | 0.132 |
|  |  |  | Prenatal × Postnatal | (1, 20) | 0.001 | 0.981 |
|  | cFos-positive cell density (number/mm^3^), pPir layer 2 | 2 × 2 (Prenatal × Postnatal) | Prenatal | (1, 20) | 9.670 | **0.006** |
|  |  |  | Postnatal | (1, 20) | 3.776 | 0.066 |
|  |  |  | Prenatal × Postnatal | (1, 20) | 0.083 | 0.777 |
|  | cFos-positive cell density (number/mm^3^), total layer 3 | 2 × 2 (Prenatal × Postnatal) | Prenatal | (1, 20) | 0.012 | 0.914 |
|  |  |  | Postnatal | (1, 20) | 2.296 | 0.145 |
|  |  |  | Prenatal × Postnatal | (1, 20) | 0.254 | 0.620 |
|  | cFos-positive cell density (number/mm^3^), aPir layer 3 | 2 × 2 (Prenatal × Postnatal) | Prenatal | (1, 20) | 0.135 | 0.717 |
|  |  |  | Postnatal | (1, 20) | 2.950 | 0.101 |
|  |  |  | Prenatal × Postnatal | (1, 20) | 0.703 | 0.412 |
|  | cFos-positive cell density (number/mm^3^), pPir layer 3 | 2 × 2 (Prenatal × Postnatal) | Prenatal | (1, 20) | 0.416 | 0.526 |
|  |  |  | Postnatal | (1, 20) | 0.951 | 0.341 |
|  |  |  | Prenatal × Postnatal | (1, 20) | 0.001 | 0.974 |
| **HPLC** | DA (ng/mg protein) | 2 × 2 (Prenatal × Postnatal) | Prenatal | (1, 14) | 0.437 | 0.519 |
|  |  |  | Postnatal | (1, 14) | 1.190 | 0.294 |
|  |  |  | Prenatal × Postnatal | (1, 14) | 4.669 | **0.049** |
|  | DOPAC (ng/mg protein) | 2 × 2 (Prenatal × Postnatal) | Prenatal | (1, 14) | 0.138 | 0.715 |
|  |  |  | Postnatal | (1, 14) | 1.126 | 0.307 |
|  |  |  | Prenatal × Postnatal | (1, 14) | 0.965 | 0.343 |
|  | DOPAC/DA (ratio) | 2 × 2 (Prenatal × Postnatal) | Prenatal | (1, 14) | 6.241 | **0.026** |
|  |  |  | Postnatal | (1, 14) | 2.474 | 0.138 |
|  |  |  | Prenatal × Postnatal | (1, 14) | 4.002 | 0.065 |
|  | 5-HT (ng/mg protein) | 2 × 2 (Prenatal × Postnatal) | Prenatal | (1, 13) | 0.018 | 0.896 |
|  |  |  | Postnatal | (1, 13) | 1.607 | 0.227 |
|  |  |  | Prenatal × Postnatal | (1, 13) | 1.613 | 0.226 |
|  | 5-HIAA (ng/mg protein) | 2 × 2 (Prenatal × Postnatal) | Prenatal | (1, 13) | 0.879 | 0.366 |
|  |  |  | Postnatal | (1, 13) | 0.197 | 0.665 |
|  |  |  | Prenatal × Postnatal | (1, 13) | 4.589 | 0.052 |
|  | 5-HIAA/5-HT (ratio) | 2 × 2 (Prenatal × Postnatal) | Prenatal | (1, 13) | 0.556 | 0.469 |
|  |  |  | Postnatal | (1, 13) | 1.348 | 0.266 |
|  |  |  | Prenatal × Postnatal | (1, 13) | 0.055 | 0.819 |

***Table S3.*** Summary of ANOVA results for the histological analyses and HPLC conducted on adult offspring in the four experimental groups (data corresponding to main *Figure 5*). The table specifies the F values for each analyzed parameter, along with the corresponding degrees of freedom (DF). Significant effects (*p* < 0.05) are given in bold font. *n* = 5-7 per group for cFos analysis; *n* = 4-5 per group for HPLC analysis.

**SUPPLEMENTARY FIGURES.**

**
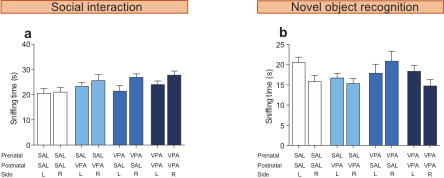
**

**Figure S1. Animals show normal responses to novel objects. a** Time spent investigating the two empty cylinders in the habituation phase of the social interaction test. No differences were observed between groups: Two-way ANOVA, prenatal treatment: *F*_1, 75_ = 3.409, *p* = 0.0688; postnatal treatment: *F*_1, 75_ = 3.828, *p* = 0.0541; interaction: *F*_1, 75_ = 0.445, *p* = 0.5059. **b** Time spent investigating the two identical objects in the training session of the novel object recognition test. No differences were observed between groups: Two-way ANOVA, prenatal treatment: *F*_1, 74_ = 0.171, *p* = 0.680; postnatal treatment: *F*_1, 74_ = 3.351, *p* = 0.0712; interaction: *F*_1, 74_ = 0.059, *p* = 0.809. Position of the cylinders or objects: L, left; R, right. Graphs indicate means + s.e.m.


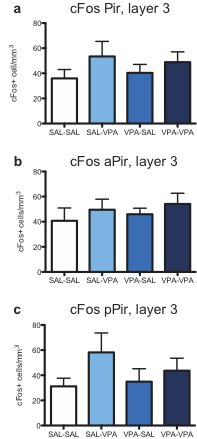


**Figure S2. Animals show similar numbers of cFos-positive cells in the layer 3 of the piriform cortex.** Density of c-Fos-positive nuclei was measured in the whole layer 3 of the Pir (a), the anterior part of the layer 3 of the Pir (b, aPir) and the posterior part of the layer 3 of the Pir (c, pPir). *n* = 5-7 mice/group. Detailed statistical information is available in Supplementary table 3.
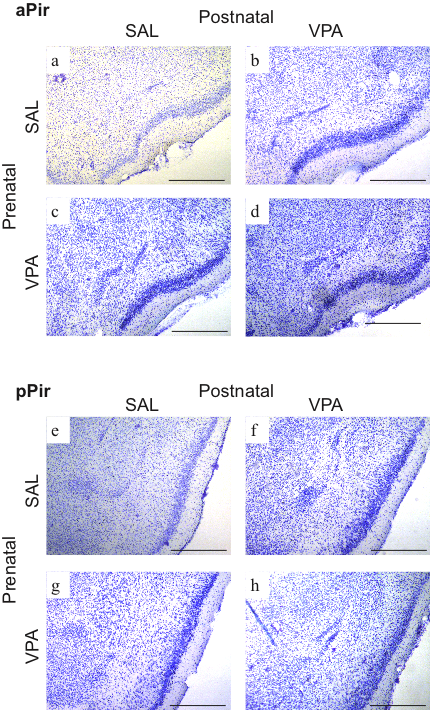
**Figure S3. Representative images of sections processed for c-Fos immunoreativity and Nissl staining. (a-d)** aPir and **(e-h)** pPir. Bars, 0.5 mm.
